# The foot drawing method: reliability of measuring foot length and outward rotation in children with clubfoot

**DOI:** 10.1186/s12891-022-05465-9

**Published:** 2022-05-28

**Authors:** Evgenia Manousaki, Hanneke Andriesse, Gunnar Hägglund, Axel Ström, Anna-Clara Esbjörnsson

**Affiliations:** 1 Department of Clinical Sciences Lund, Orthopedics, Lund University, Skane University Hospital, 221 85 Lund, Sweden; 2 Department of Orthopedics, Central Hospital in Växjö, Växjö, Sweden; 3 Clinical Studies Sweden Forum South, Skane University Hospital, Lund, Sweden

**Keywords:** Clubfoot, Foot length, Foot rotation, Reliability

## Abstract

**Background:**

The Ponseti method is the gold standard for clubfoot treatment. However, relapse and residual gait deviations are common, and follow-up until 7 years of age is recommended. We evaluated the reliability of the foot drawing method, a new instrument for the follow-up of clubfoot. The method uses drawings of the foot in the neutral position and external rotation to measure foot length and outward rotation.

**Methods:**

Nineteen children aged 2.5–7 years who were treated with the Ponseti method for congenital clubfoot were included. Two raters made the drawings twice (D1 and D2). Each rater measured foot length, foot rotation, and foot–tibial rotation independently (D1). Later, the raters repeated the measurements (D2). Interrater reliability was assessed using the D1 from each rater. Intrarater reliability was assessed using the measurements from each rater’s D1 and D2. Bland–Altman plots were used to visualize the limits of agreement (LoA). The mean, 95% confidence interval, and one standard deviation of the differences in all measurements were calculated.

**Results:**

The mean differences between and within raters were: foot length < 1 mm, foot rotation < 1°, and foot–tibia rotation < 2°, which indicated no systematic differences. The LoA for foot length were: 4.5 mm and 5.9 mm between raters for D1, − 4.8 mm and 5.9 mm for rater 1 (D1–D2), and − 5.1 mm and 5 mm for rater 2 (D1–D2). The LoA for foot rotation: were − 12° and 10.6° between raters (D1), − 8.4° and 6.6° for rater 1 (D1–D2), and − 14° and 14.1° for rater 2 (D1–D2). The LoA for foot–tibia rotation were: − 17.8° and 14.3° between raters (D1), − 12° and 12.2° for rater 1 (D1–D2), and − 12.7° and 13.6° for rater 2 (D1– D2).

**Conclusions:**

The absence of systematic differences between and within raters, and LoA observed indicate that the foot drawing method is applicable in clinical practice and research. However, the results of the foot and foot–tibia rotation analyses imply that caution is needed when interpreting changes in foot rotation in feet with higher degrees of rotation.

**Supplementary Information:**

The online version contains supplementary material available at 10.1186/s12891-022-05465-9.

## Background

The Ponseti method is considered to be the gold standard for clubfoot treatment [1–3]. However, relapse and residual gait deviations are common, even after successful initial treatment [3–5], and children with clubfoot are recommended to undergo clinical follow-up at least until the age of 7 years [6]. Different measures are used to evaluate clubfoot status [7–13], but there is a lack of agreement about which measurements are the most appropriate [14].

A recent study of foot length (FL) in children with clubfoot showed that feet with clubfoot normally grow regularly over time and that feet with a shorter length than expected are more susceptible to relapse [15]. Thus, a reliable method of measuring the FL is of clinical importance.

Since 1995, a continuous follow-up of all children with clubfoot is performed at our department according to a standardized protocol [16]. To this standardized protocol, drawings on paper of the foot in a neutral position and externally rotated were added. The drawings were used to measure FL and foot outward rotations and provide a simple visual method for evaluating the development of FL and mobility. We named this novel procedure the foot drawing method (FDM).

To our knowledge, no studies have reported on the development of foot rotations in children treated for clubfoot. Foot abduction (called foot rotation from this point) is defined by Ponseti as the foot’s ability to rotate outward and is a key correction parameter [2]. Casting-and-stretching techniques are intended to mobilize the clubfoot, and orthotic treatment is used to maintain mobility [2]. Foot rotation depends on the mobility of the tibiotalar and subtalar joints in combination with mid-and forefoot abduction. In addition, rotation in the knee and hip joints, as well as tibia and femur torsion may influence foot rotation. Regular assessment of the range of foot rotation may provide a simple method for detecting changes in mobility. As far as we know, there is no method that assess the changes in foot and tibia rotation in children with clubfeet.

The FDM is a simple and cheap instrument that measures FL and foot outward rotations in children with clubfoot. The aim of this study was to evaluate the intra- and interrater reliability of the FDM, in children with clubfeet treated with the Ponseti method.

## Patients and methods

### Patients

The study is a reliability study. It was approved by the National Ethical Review Authority (Dnr 2020–03008). Only children with idiopathic congenital clubfoot treated at our department were included. The children were already scheduled to have their usual follow-up visits. Twenty children were invited, and all agreed to participate in the study. The children were 2.5–7 years of age and were scheduled to have their usual follow-up visits during October and November 2020. One child canceled the visit because of illness. All parents were verbally informed in advance about the procedures and provided written informed consent. All children were treated according to the Ponseti method [2]. Foot abduction orthosis (FAO) was used until at least the age of 4 years and longer if indicated [3].

### Methods

#### The foot drawing method (FDM)

##### Foot drawing

**Fig. 1:**
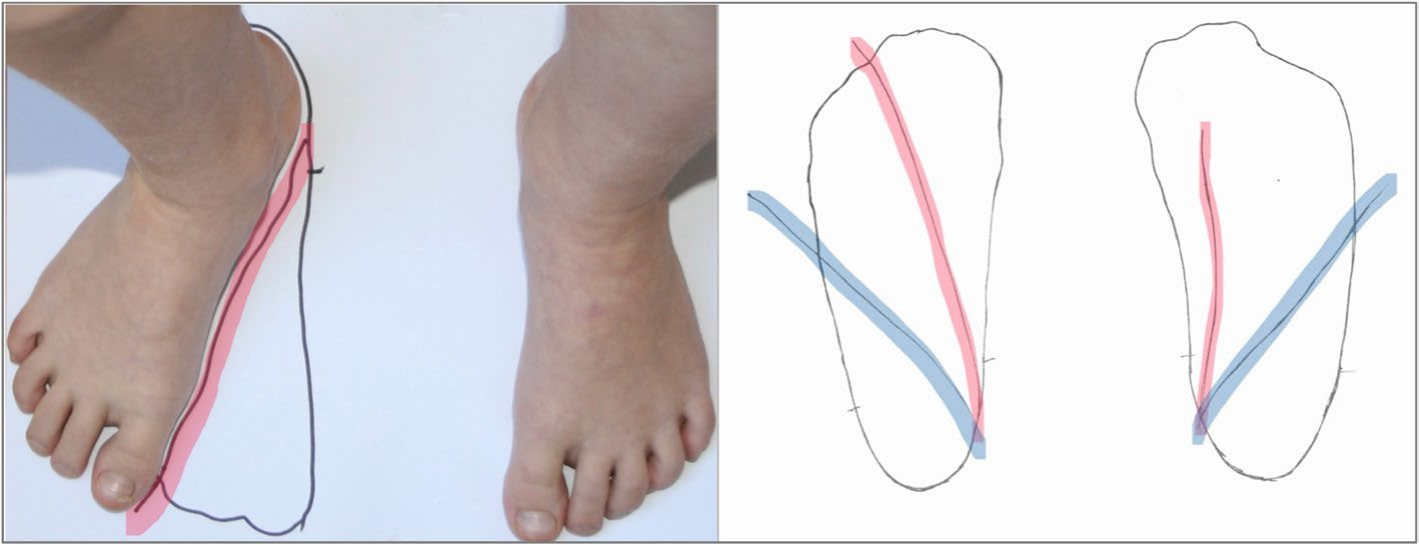
A line is drawn around each foot with the pen kept vertical. Two more lines are added. The first line marks the medial foot edge after the foot has been rotated in maximal external rotation in relation to the tibia (red line). The second marks the medial foot edge after the foot has been rotated further outward on the knee level by adding maximal tibial rotation (blue line)

The child sits on a chair with the hips and knees in 90° of flexion, and the full foot in contact with the floor. The examiner sits in front of the child and places a paper on the floor parallel to the front of the chair. The examiner holds the foot lightly on the outside with one hand and moves the lower leg into flexion and extension in the knee. The other hand adjusts the thigh so that the tibial tuberosity is pointing straight forward. The foot is then placed down on the paper in its natural position [12, 15]. A line is drawn around the foot holding the pen vertically, and the medial and lateral malleoli are marked on the paper. Then, while holding the lower leg with one hand, the examiner rotates the foot into maximal outward rotation under the talus in the subtalar joint while fixating the talus as in Ponseti’s abduction movement. The medial foot margin is then drawn again. Thereafter, the examiner fixes the distal femur. The foot is then rotated further outward with the other hand, which induces tibial–fibular outward rotation at the knee level. The medial foot margin is marked again (Fig. 1). Both feet are drawn using this procedure, even the contralateral foot in unilateral cases.

##### Foot measurements

The foot length (FL) and outward rotations are measured with ruler and goniometer directly on the piece of paper where the footprint is drawn. The measurements are performed as follows.

**Fig. 2:**
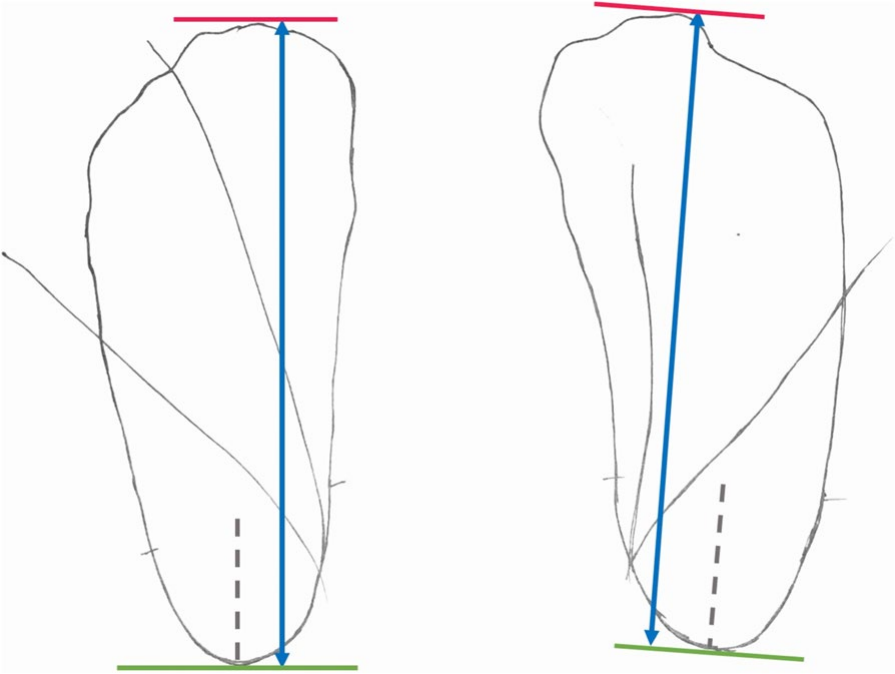
Foot length (blue line) is defined as the distance between two lines. The proximal line (green) is perpendicular to the imaginary line that passes from the middle of the hindfoot (grey line). The distal line (red) is parallel to the proximal line and includes the whole footprint

**Fig. 3:**
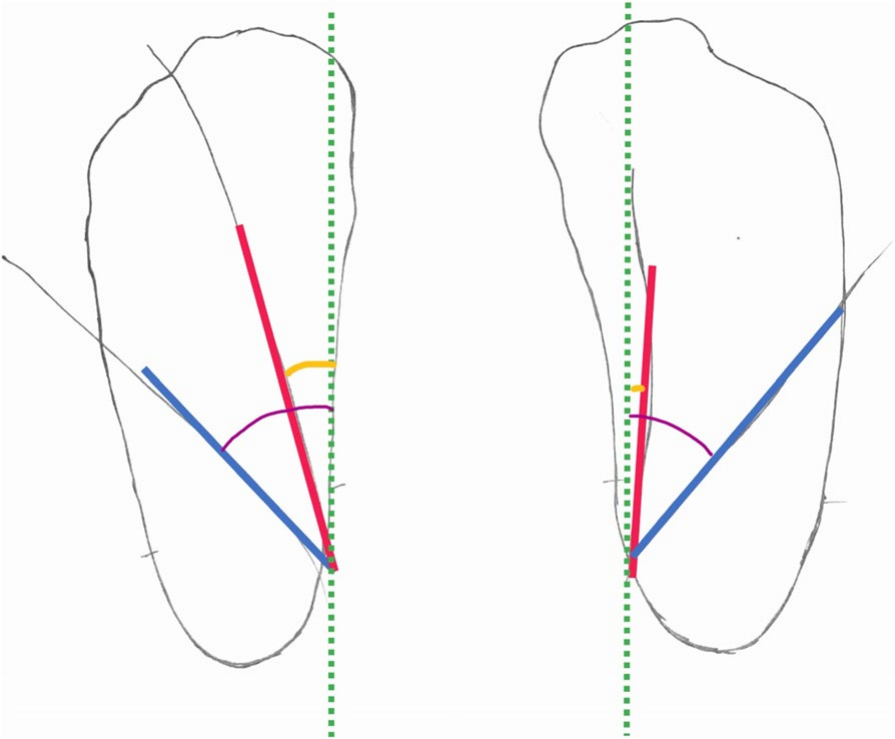
Foot rotation (yellow) and foot and tibia rotation (FTR, purple) are measured with a goniometer between each foot margin (red: FR, blue: FTR) and a line (green) drawn vertically to the long side of the paper

- *Foot length:* FL is measured as described by Manousaki et al. in 2021 [15] as the distance between two parallel lines, drawn distally and proximally on the footprint: The proximal line is perpendicular to an imaginary line passing through the middle of the hindfoot. The distal line is parallel to the proximal line. The 2 parallel lines should include the whole footprint, regardless the shape or the anatomical variation of the footprint (Fig. 2).
- *Foot outward rotation (FR):* we defined FR as the angle between the medial foot margin (not including forefoot adduction or abduction) with the foot in maximal outward rotation as described above and a line drawn vertically to the long side of the paper (Fig. 3). The angle is measured with a goniometer (Fig. 3).
- *Foot and tibial–fibular outward rotation (FTR):* we defined FTR as the angle between the medial foot margin with the foot and lower leg in maximal outward rotation as described above and the line drawn vertically to the long side of the paper. The angle is measured with a goniometer (Fig. 3).

### Procedures

The intra and inter-reliability for the whole drawing procedure between 2 experienced examiners, rater 1 and rater 2 was assessed. Rater 1 and rater 2 made drawings (Ds) of the children’s feet twice (D1 and D2), independently of each other. A short break between each drawing was taken to allow the child to walk around. First, one rater made both drawings, and then, after a short break, the other rater repeated the procedure. The starting order of raters alternated for every new patient.

Each of the two raters scanned their drawings (D1 and D2) and measured FL, FR, and FTR directly on the copy of their first drawing (D1). Thereafter both the originals and copies were given to an independent person. After a period of 3–4 weeks, the raters received their D2 drawing, which was anonymized, and they repeated the measurements of FL, FR, and FTR. These were collected by an independent person and forwarded to another independent person for further analysis.

### Statistical analysis

Power analysis was performed for both FL and foot rotations to estimate the number of feet needed to identify systematic differences. For the power analysis, we estimated a priori that, to detect clinically significant systematic differences between assessments, paired t-tests would be used. Further differences of 4 mm and a standard deviation (SD) of 2 mm in FL and 5° (SD 3°) in foot rotations would be assumed. The measurements of foot rotation required more feet, at least seven, to investigate systematic differences with a power of 80% and α = 5%. To study the possible effects of age, we chose to have two age groups (4.5 years or younger and older than 4.5 years of age). Therefore, we decided to include at least 14 children but increased this further to account for dropouts.

The D1 from each rater was used to assess interrater reliability. The measurements from each rater’s D1 and D2 were used to assess intrarater reliability. Bland–Altman plots were used to visualize the limits of agreement (LoA). The mean, 95% confidence interval (CI), and SD of the differences were calculated for all measurements. Independent samples t-test was used to compare the foot rotation measurement results from the two different age groups. Differences were considered statistically significant for *p*-values < 0.05.

Both feet, with and without clubfoot for children with unilateral involvement, were included in the analysis. Before including all feet, we performed a sensitivity analysis, in order to determine if all feet, even the unaffected feet in unilateral clubfeet could be included. First, the statistical analysis was performed including unilateral clubfeet and randomly one clubfoot from each of the children with clubfoot in both feet. Thereafter the analysis was performed including all clubfeet but excluded the contralateral unaffected foot in participants with unilateral clubfoot. Finally, the analysis was performed including all feet. The results from the two different approaches (one foot from each child vs one foot from those with unilateral clubfoot and two feet from those with bilateral clubfoot) were similar to the results from the analysis that included all feet. Hence, the results presented in this article include both feet from all included children.

All analyses were performed using R version 3.6.3 [17].

## Results

Nineteen children (15 boys, 12 with unilateral clubfoot) aged 2.5–7 years were included. Eleven children were older than 4.5 years and eight children were 4.5 years or younger. The average FR was greater for children aged ≤4.5 years (21° (SD 6°)) than for those > 4.5 years (15° (SD 5°)) (*p* = 0.001). The average FTR was also greater for the younger children (52° (SD 13°)) than the older children (40° (SD 10°)) (*p* = 0.005). The average FR for the contralateral unaffected feet (all ages) was 19° (SD 3°), and the average FTR was 48° (SD 7°). Increased intra- and interrater differences were observed with increasing degrees of rotation for FR and FTR. Neither age nor clubfoot laterality had an effect on these differences. Thus, we present the following results for the entire group without dividing the children into age groups.

The mean difference between and within raters was < 1 mm for FL, < 1° for FR, and < 2° for FTR (Tables 1 and 2). These small differences did not differ statistically or clinically.

**Table 1:** Mean differences between each rater’s first measurement D1 (interrater reliability)

Measurement	Mean (95% CI)	SD	LoA
Foot length (mm)	0.7 (− 0.2; 1.6)	2.6	−4.5; 5.9
Foot rotation (°)	−0.7 (−2.6; 1.2)	5.8	−12; 10.6
Foot–tibia rotation (°)	−1.7 (−4.4; 0.7)	8.2	−17.8; 14.3

*CI* Confidence interval, *SD* Standard deviation, *LoA* Limits of agreement

**Table 2:** Mean differences between first and second measurements for each rater

Measurement	Intrarater R1			Intrarater R2		
	Mean (95% CI)	SD	LoA	Mean (95% CI)	SD	LoA
Foot length (mm)	−0.6 (− 1.5; 0.3)	2.7	− 5.9; 4.8	0 (− 0.8; 0.9)	2.6	−5; 5.1
Foot rotation (°)	−0.9 (−2.2; 0.4)	3.8	−6.6; 8.4;	0.1 (−2.3; 2.4)	7.2	− 14; 14.1
Foot–tibia rotation (°)	0.1 (−1.9; 2.1)	6.2	−12; 12.2	0.4 (− 1.8; 2.7)	6.7	− 12.7; 13.6

### Foot length

**Fig. 4:**
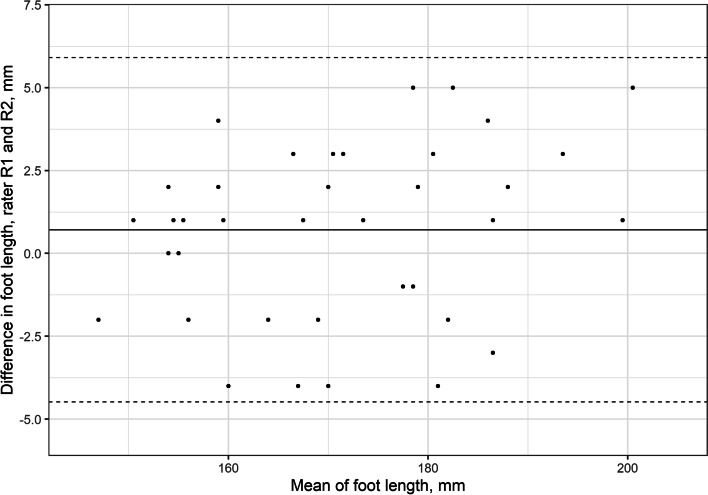
Interrater Bland–Altman plot for foot length. The y-axis shows the difference between the first (D1) measurements of the two assessors. The x-axis shows the mean foot length

The LoA for FL between raters for the first measurement (D1) were − 4.5 mm to 5.9 mm (Table 1, Fig. 4). The intrarater LoA between D1 and D2 were − 4.8 mm to 5.9 mm for rater 1 and − 5.1 mm to 5 mm for rater 2 (Table 2).

*R1* Rater 1, *R2* Rater 2, *CI* Confidence interval, *SD* Standard deviation, *LoA* Limits of agreement.

### Foot outward rotation

**Fig. 5:**
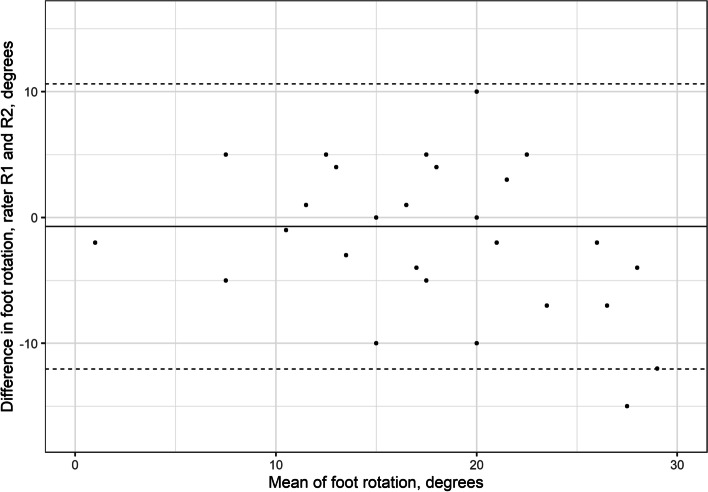
Interrater Bland–Altman plot for foot rotation. The y-axis shows the difference between the first measurement (D1) of the two assessors. The x-axis shows the mean foot rotation

The LoA for FR between raters’ D1 were − 12° to 10.6° (Table 1, Fig. 5). The intrarater LoA were − 6.6° to 8.4° for rater 1 and − 14.1° to 14° for rater 2 (Table 2).

### Foot and tibia–fibular outward rotation

**Fig. 6:**
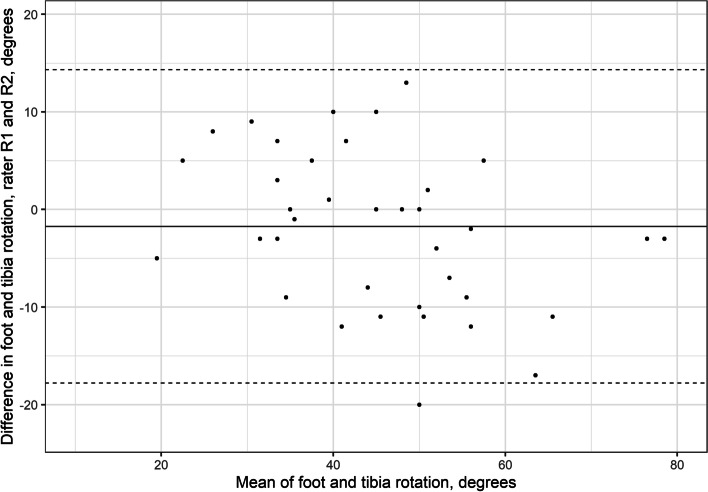
Interrater Bland–Altman plot for foot and foot-tibia rotation. The y-axis shows the difference between the first (D1) measurement of the two assessors. The x-axis shows the mean foot-tibia rotation

The LoA for FTR between raters’ D1 were − 17.8° to 14.3° (Table 1, Fig. 6). The intrarater LoA were − 12° to 12.2° for rater 1 and − 12.8° to 13.6° for rater 2 (Table 2).

## Discussion

We evaluated the reliability of the FDM, a new instrument for follow-up of children with clubfoot, in 19 children treated according to the Ponseti method. The systematic differences between raters were small (< 1 mm for FL and < 2° for FR and TR), which indicated that no significant bias was observed. The LoA was < 6 mm for FL and < 18° for FR and FTR. The intra- and interrater differences were more pronounced in feet with higher degrees of rotation.

Different methods to measure FL have been reported as reliable [7, 9]. In our study, the small mean differences (< 1 mm (SD < 3 mm)) between measurements and between and within raters indicate that FL could be used in research to compare different treatment groups. The Bland–Altman plots for FL showed good intra- and interrater LoA (Table 1, Fig. 4). Until the age of 7 years, feet with clubfeet grow about 1.2 cm per year [15]. The LoA observed in the Bland–Altman analysis indicate that changes > 6 mm are clinically detectable. This implies that the FL could be useful for follow-up as the child grows. A previous study showed that FL at the age of 2 years could be used as a prognostic tool as children with unilateral clubfoot with smaller FL compared with the contralateral foot at 2 years of age had a higher tendency to relapse and poorer motion quality at 7 years of age [15]. Small clubfeet are also associated with difficulties at the initial correction and a higher tendency to relapse [10, 11, 18].

To our knowledge, the longitudinal development of FR and FTR in children with clubfoot has not been investigated. Gupta et al. [19] showed that, in healthy children aged 0–8 years, foot abduction (external rotation of the foot, with flexed knee, simulating the Ponseti maneuver) ranged from 20° to 101° (mean 61° (SD 14°)). Outward rotation of the clubfoot is important during the initial correction with Ponseti casting. After the initial correction, the acquired outward rotation (i.e., abduction) is maintained with the FAO by positioning the feet in 60° of outward rotation. This outward rotation is a combination of mobility from different joint levels such as foot, ankle-tibia, knee, and even hip joint. The older children in our study had significantly smaller FR and FTR angles. The use of FAO generally ends by the age of 4 years, and the lower outward rotations found in older children in this study may reflect this. Further studies on foot-tibia rotational development in relation to treatment could be of interest.

The estimated error in goniometer measurements used for clinical assessments range from 5° to 25° [20–23]. The variations depend on the body segment measured, procedure, and experience of the examiners [20–24]. Intrarater reliability is usually better than interrater reliability. The small mean differences for the FR and FTR measurements (< 2° (SD 4°)) and the absence of systematic bias found in our study indicate that the method may be useful in research involving group comparisons. The LoA according to Bland–Altman analysis were relatively high: up to 14° for intrarater and 18° for interrater reliability. Nevertheless, the Bland–Altman plots showed that more pronounced differences were observed when the FR and FTR angles were high (> 20° for FR and 45° for FTR, Figs. 5 and 6). This implies the presence of proportional bias. Because most functional problems appear when the feet are becoming stiff (i.e., developing less rotation), this bias has little impact on the usefulness of the FDM to measure clinically important ranges of motion. However, these pronounced differences between and within raters require further evaluations. In the clinical settings it is advised to compare with previous measurements, to double check the results.

Several sources of error may have contributed to the differences observed, for example, the positioning of the foot and the force each rater used to rotate the foot. Additionally, the child’s cooperation, ability to understand instructions, and attention span may influence reliability. Other factors that are important to consider when testing reliability are sample size, statistical method, assessment procedure, and experience of the assessors. The FDM comprises three procedures that all affect reliability: positioning of the foot on the paper, drawing around the foot, and measuring the parameters. When the FDM is used clinically, all procedures are completed during the visit. Thus, we chose not to divide the procedures in this reliability analysis.

Bland–Altman plots were used for evaluating the reliability of the FDM. In comparison with Intraclass Correlation Coefficient (ICC), commonly used in reliability studies, Bland–Altman plots have the advantage of visually presenting the differences of all individual measurements as well as any systematic differences and the Level of Agreement is expressed in mm or degrees and not as a correlation coefficient. Hence, Bland–Altman plots are more valuable when evaluating a method for the first time.

We included both feet from each child, even the unaffected foot for children with unilateral clubfoot. Because the feet from the same child are related and the aim of our study was to analyze the reliability of the method and not the outcome of a treatment, we felt it was important to include both feet. In the clinical setting, the appearance and mobility of both feet, including the unaffected in children with unilateral clubfoot, are drawn and analyzed. One limitation of the study was that both raters had long experience with the FDM and with clubfoot treatment, both of which can affect reliability [24]. By standardizing the method as described previously, we expect that less experienced colleagues will be able to use the method. The reliability of the method with inexperienced examiners is yet to be determined.

## Conclusions

We conclude that the novel FDM is applicable in clinical practice and research in the context for which it is aimed to be used. By comparing measurements during follow-up, developments in foot growth and foot mobility can be monitored. However, the results from the foot and foot–tibia rotation analysis imply that the method is more reliable in stiff feet and caution is needed when interpreting changes in foot rotation in feet with higher degrees of rotation.

## Supplementary Information

**Additional file 1.**

## Data Availability

All data generated or analyzed during this study are included in this published article and its supplementary information files.

## Abbreviations

D1: Drawing 1
D2: Drawing 2
FL: Foot length
FDM: Foot drawing method
FR: Foot outward rotation
FTR: Foot and tibial–fibular outward rotation
SD: Standard deviation
LoA: Limits of agreement
CI: Confidence interval
ICC: Intraclass correlation coefficient
